# Evolution of Neuroaesthetic Variables in Portrait Paintings throughout the Renaissance

**DOI:** 10.3390/e22020146

**Published:** 2020-01-26

**Authors:** Ivan Correa-Herran, Hassan Aleem, Norberto M. Grzywacz

**Affiliations:** 1 Department of Neuroscience, Georgetown University, Washington, DC 20057, USA; iac16@georgetown.edu; 2 Facultad de Artes, Universidad Nacional de Colombia, Bogotá 110111, Colombia; 3 Interdisciplinary Program in Neuroscience, Georgetown University, Washington, DC 20057, USA; ha438@georgetown.edu; 4 Department of Physics, Georgetown University, Washington, DC 20057, USA; 5 Graduate School of Arts and Sciences, Georgetown University, Washington, DC 20057, USA

**Keywords:** neuroaesthetics, symmetry, balance, complexity, chiaroscuro, normalized entropy, renaissance, portrait paintings, art history, art statistics

## Abstract

To compose art, artists rely on a set of sensory evaluations performed fluently by the brain. The outcome of these evaluations, which we call neuroaesthetic variables, helps to compose art with high aesthetic value. In this study, we probed whether these variables varied across art periods despite relatively unvaried neural function. We measured several neuroaesthetic variables in portrait paintings from the Early and High Renaissance, and from Mannerism. The variables included symmetry, balance, and contrast (chiaroscuro), as well as intensity and spatial complexities measured by two forms of normalized entropy. The results showed that the degree of symmetry remained relatively constant during the Renaissance. However, the balance of portraits decayed abruptly at the end of the Early Renaissance, that is, at the closing of the 15th century. Intensity and spatial complexities, and thus entropies, of portraits also fell in such manner around the same time. Our data also showed that the decline of complexity and entropy could be attributed to the rise of chiaroscuro. With few exceptions, the values of aesthetic variables from the top of artists of the Renaissance resembled those of their peers. We conclude that neuroaesthetic variables have flexibility to change in brains of artists (and observers).

## 1. Introduction

Aesthetic emotions are not arbitrary. For instance, people exhibit aesthetic preference for visual art with high degrees of symmetry across many cultures [1,2]. Other such visual properties with universal impact on aesthetic values are balance, contrast, and complexity (measured as normalized entropy—[3,4]. Why do these properties have universal aesthetic impact? The Processing Fluency Theory provides a simple answer by postulating that sensory variables processed by the brain with ease facilitate positive aesthetic emotions [5,6]. Hence, if the brain has specialized mechanisms to deal with a sensory variable, it will tend to be aesthetically valuable. This is the case for symmetry, balance, contrast, and complexity variables, which have dedicated neural circuitries, because of their evolutionary importance. We call such properties like symmetry, balance, contrast, and complexity neuroaesthetic (or fluency) variables, since their importance emerges directly from neural constraints [7].

Because specialized brain mechanisms constrain neuroaesthetic variables, one may expect that they remain relatively constant over time, especially across art periods [8]. However, a recent study found that artists exhibited an appropriate bias towards these variables, but did not optimize them. Moreover, artists also exhibited individuality with respect to these variables [4]. Because of this individuality, a certain degree of flexibility appears to exist with respect to neuroaesthetic variables. Therefore, they could potentially evolve across different periods of art. The possibilities that neuroaesthetic variables could either remain constant or evolve across art periods raised a series of questions in our minds: Are changes in art periods occurring in the absence of evolution of neuroaesthetic variables? Conversely, if these variables evolve over time, in what directions are the changes? For example, would the degrees of symmetry, balance, contrast, and complexity necessarily increase over time following the improvement of artistic techniques? And would the evolution in neuroaesthetic variables at the boundaries of different art periods (as determined by art historians) be abrupt? So far, there has been limited research aimed at answering such questions. One notable study looked at changes in fractal dimension and Shannon entropy in western paintings ranging from years 1285 to 2008 [9]. The research found that both measures remained relatively stable over time, except for an abrupt change around the late nineteenth century. The author speculates that this change may indicate the transition from pre-Modern to Modern Art.

In the study reported here, we probed what happened to the neuroaesthetic variables across the three periods of the Renaissance. These periods were the Early and High Renaissance, and the Mannerism (Late Renaissance—[8]). Art historians have characterized the differences between these art periods in terms of key artistic concepts. New concepts were continuously discovered or rediscovered during the Renaissance, and introduced in the work of artists. For example, Alberti’s books on painting [10] and architecture [11] introduced new concepts that influenced the theory of the arts during the Renaissance itself. These concepts included ideas that evolved throughout the Renaissance, such as harmony, golden ratio, naturalism, anatomical studies, linear perspective, aerial perspective, and *chiaroscuro* [8]. These ideas are related to the neuroaesthetic variables mentioned above. Harmony and golden sections have to do with balance and symmetry. In turn, naturalism, anatomical studies, and the two forms of perspective produce realism and thus, complexity. Finally, *chiaroscuro* (translates to ‘bright and dark’ in Italian) is related to contrast. In *chiaroscuro*, strong tonal contrast between light and dark in different regions of a painting helps highlight its important parts, often with a dramatic effect. Furthermore, *chiaroscuro* helps to model three-dimensional forms through shades. Consequently, *chiaroscuro* along with the other concepts were elements of a new theory that transformed art from the practices of the Middle Ages.

Finally, our study focused on portrait paintings during these three periods. Our rationale for focusing on portraits was their relative simplicity, as they centered solely on the depiction of the human subject as opposed to other forms of art. In addition, portraits tended to have a vertical composition, simplifying the measurement of symmetries in the canvas. Therefore, focusing on portraits helped us constrain our study in a simpler set of measurements and statistics. Another reason to focus on portraits was that they encouraged interesting evolutionary tendencies across time during the Renaissance. Such evolution happened because portraits set up a competition among artists and their workshops to gain the favor of patrons and get the commissions [12]. This competition led the painters to explore new artistic forms to represent the character of the individual subject. Thus, portrait paintings evolved and improved over time.

In this article, we begin with a series of statistical measurements on symmetry, balance, and complexity. We chose these variables because they have a direct relationship to processing fluency [13,14]. In this study, we expand the measurements to all three periods of the Renaissance and compare Italy with the rest of Europe. We also attempt to compare the most famous painters of those times (as judged today) with other Renaissance painters. This comparison allows studying whether these two cohorts of painters differ significantly in terms of neuroaesthetic variables. Finally, some of the findings related to the evolution of balance and complexity were surprising. We attempted to explain them statistically through both the emergence of perspective and new measurements of the degree of *chiaroscuro*.

## 2. Materials and Methods

Methods for image selection, and measurements of symmetry, balance, and complexity appear in detail elsewhere [4]. In this section, we mainly focus on methods that are unique to this article. New qualitative methods include the choice of portrait paintings (Section 2.1), selection of top portrait painters of the Renaissance (Section 2.2), and classification of paintings into stylistic characteristics (Section 2.3). New quantitative methods are the statistical analyses (Section 2.4) and the development of indices of *chiaroscuro* (Section 2.5). In Section 2.6, we rewrite the equations of symmetry, balance, and complexity in [4] using the notation of Section 2.5.

### 2.1. Portrait Paintings

We studied 456 portrait paintings from 53 painters. We only included a painting in the study if it displayed one main individual as the subject. The portraits were painted in oil, tempera, frescos, or a mixture of these materials. We obtained all paintings from the “Artstor Digital Library” (library.artstor.org). If the painting had a frame, we removed it before performing our analyses, except if the painter had painted it. While all images were originally in color, we converted them into an 8-bit grayscale by rounding the average of the red, green, and blue values.

A complete list of the painters, paintings (by Artstor file name), and their classification into periods and stylistic characteristics appears in the Supplementary Materials. To each painting, we assigned a date of completion, giving the median values in case art historians are uncertain about the exact times. We excluded paintings with more than 50 years of uncertainty from the study. We decided to allow such an uncertainty in the determination of the time of completion, because such uncertainty happened rarely and we used robust statistics for all our conclusions (Section 2.4). The classification into Early Renaissance, High Renaissance, and Mannerism used the date of birth of the painters. Thus, we took painters of the Early Renaissance as those born between 1370 and 1450. In turn, painters of the High Renaissance were born between 1452 and 1489. Finally, Mannerists were those born from 1494 until 1571. This manner of classification into periods can be debated, especially at their borders. However, as shown in Supplementary Materials, the classification yields results accepted by art historians [8]. Furthermore, border errors were removed by the robust statistics in our articles (Section 2.4).

### 2.2. Selection of Top Portrait Painters

We wished to compare the most renowned painters of the Renaissance (as judged today) with other good painters from the same period. We know of no objective way to make a list of the most famous artists and different people may disagree on it. However, some rankings do exist and we decided to use one of them. We used the ranking developed by Ranker, a digital media company that produces polls on entertainment, brands, sports, and culture (Ranker.com). The list that Ranker has produced on Renaissance artists appears in http://bit.ly/RankerRenaissance (accessed 11 April 2018). Table 1 shows the top-ten vote getters in this list on 11 April 2018:

As the table indicates, we used seven of these ten artists in our study. The other three were renowned for other forms of art not portraits. Further support that these seven portrait artists are among the leading ones from the Renaissance comes from [15].

### 2.3. Classification of Paintings into Stylistic Characteristics

To try understanding some surprising findings related to the evolution of balance and complexity, we divided the portrait paintings into four categories, namely, *chiaroscuro*, linear perspective, aerial perspective, and none of those. This classification was performed by eye by one of us, ICH. He used the following definitions for the classification:

- Linear Perspective: perspective in which the relative size, shape, and position of architectural objects are determined by imagined lines converging at a point on the horizon.
- Aerial Perspective: the technique of representing distant objects as fainter and bluer.
- *Chiaroscuro*: an effect of contrasted light and shadow created by light falling unevenly on something.

Fortunately, no portrait painting seemed to belong to more than one of these categories.

### 2.4. Statistical Analyses

All analyses comparing neuroaesthetic variables across locations and art periods used two-way ANOVA followed by post-hoc two-sided *t*-tests. In turn, we compared the probability of artistic styles (Section 2.3) across art periods with the Fisher’s exact test. Finally, the comparison of neuroaesthetic variables for different artistic styles employed one-way ANOVA followed by post-hoc one-sided *t*-tests.

We performed tests of temporal trends of neuroaesthetic variables with the robust Kendall’s τ correlation coefficient. The probability that this coefficient is different from zero and estimates of error are as developed by [16]. We used the Kendall’s τ correlation coefficient, since the data exhibited outliers and trends often did not seem linear. To quantify the trends, we attempted to obtain robust fits with each of the following functions:

(1) $$\emptyset_{C}\left(t:\alpha_{1}\right)=\alpha_{1},$$

(2) $$\emptyset_{L}\left(t:\alpha_{1},\alpha_{2}\right)=\alpha_{1}+\alpha_{2}\left(t-t_{0}\right),$$

(3) $$\emptyset_{exp}\left(t:\alpha_{1},\alpha_{2},\alpha_{3}\right)=\alpha_{2}+\left(\alpha_{1}-\alpha_{1}\right)e^{-\left(t-t_{0}\right)/\alpha_{3}},$$

(4) $$\emptyset_{erf}\left(t:\alpha_{1},\alpha_{2},\alpha_{3},\alpha_{4}\right)=\alpha_{1}+\alpha_{2}\;\mathrm{erf}\left(\left(\left(t-t_{0}\right)-\alpha_{3}\right)/\left(\sqrt{2}\alpha_{4}\right)\right),$$

where t was time, $t_{0}$ was the year of the first painting in our dataset, $\alpha_{1},\alpha_{2},\alpha_{3}$ and $\alpha_{4}$ were the parameters of the functions, and *erf* was the error function [17]. Equations (1)–(4) represent constant, linear, exponential, and error-function trends respectively. The parameters of these functions have familiar interpretations. For the linear trend, $\alpha_{1}$ is the estimated value of the neuroaesthetic variable at $t_{0}$ and $\alpha_{2}$ is the slope of the change. Similarly, for the exponential trend, $\alpha_{1}$ is the estimated value of the neuroaesthetic variable at $t_{0}$. However, $\alpha_{2}$ is the value at long t’s, and $\alpha_{3}$ is the rate constant of the change. Finally, for the *erf* trend, $\emptyset_{erf}$ is a sigmoidal function [18]. Its point of fastest rate of change is $\alpha_{3}$, with $\alpha_{1}$ being the value of the function at that point. In turn, $2\alpha_{2}$ would be the output range of $\emptyset_{erf}$ if t were to vary from −∞ to +∞, with $\alpha_{4}$ setting the rate of the transition.

To obtain robust fits of these functions to the values of neuroaesthetic variables, we proceeded as follows: Let $v_{i}$ be the values of paintings completed at times $t_{i}$ where 1≤i≤N and N is the number of paintings in the dataset. To reduce the effect of outliers, we computed the M medians ${\bar{v}}_{i}$ of non-overlapping subsets of the data comprising temporally consecutive paintings. The number of paintings included in the medians ranged from 25 to 35 depending on the noise in the data. We indicate this number for each case when describing the results. We also computed the median time, ${\bar{t}}_{i}$ for each of the ${\bar{v}}_{i}$. For these pairs, we then computed:

(5) $$\vec{\alpha }={\mathrm{argmin}}_{{\vec{\alpha }}^{*}}\sum_{i=1}^{M}{\left({\bar{u}}_{i}-\emptyset_{x}\left({\bar{t}}_{i}:{\vec{\alpha }}^{*}\right)\right)}^{2},$$

where $\vec{\alpha }$ was the optimal set of parameters for each fit (e.g., $\vec{\alpha }=\left(\alpha_{1},\alpha_{2},\alpha_{3},\alpha_{4}\right)\;$for Equation (4)), and the subscript x captured one of the functions in Equations (1)–(4) (e.g., x=erf for Equation (4)). To perform this computation, we used a trust-region-reflective algorithm [19,20]. Each fit computation used five initial conditions to minimize local-minimum trapping. The initial conditions were random and chosen from the ranges of either $v_{i}$ or $t_{i}$ (the latter for variables with time dimensions). Means and standard errors of fit parameters were obtained for those initial conditions yielding errors (Equation (5)) of not more than 10% of the minimum. Although this fit was a least-squares procedure, the estimates were robust because of the median steps to obtain ${\bar{t}}_{i}$, and ${\bar{v}}_{i}$.

To probe the quality of the fits provided by Equations (1)–(4) and to compare them, we used a regression test for arbitrary fits [21,22]. In this test, a linear regression was performed in the data-versus-fitted-model scatter plot. If the model was good, this regression should be close to a straight line, with slope = 1, and intercept = 0. Consequently, the regression test probed whether the correlation coefficient, slope, and intercept were statistically significantly larger than zero, not different from 1, and not different from 0, respectively. We used the *p*-values of the tests to compare the fits (*t*-tests). The calculations of the *p*-values considered the number of parameters of the equations through the degrees of freedom (d.f.). Hence, for example, if the linear and exponential fits gave similar results, the linear one was better, because it had fewer parameters.

Finally, we tested whether particular painters behaved differently from the population for each neuroaesthetic variable. For this purpose, we used a two-sided *t*-test of whether the neuroaesthetic variable was statistically significantly above or below the optimal trend line.

For each neuroaesthetic variable, we removed outliers with a median-absolute-deviation (MAD) method [23] (MAD > 3.5) before beginning the statistical analyses.

All statistical tests, and the computations described in Section 2.5 and Section 2.6 were performed with MATLAB R2015a (MathWorks, Natick, MA, USA), using code specially developed for this project.

### 2.5. Chiaroscurro Indices

We developed two computational indices of *chiaroscuro*. We begin the description of each with a paragraph providing the physical intuition of the proposed calculations. We hope that these paragraphs will allow the reader to understand the rationale even by skipping the equations, which in turn, appear after the introductory paragraphs.

The *chiaroscuro* technique tries to use intensities close to the extremes, i.e., some regions bright and others dark. In its extreme form, *chiaroscuro* would make all the points of the painting either black or white. To measure the Index of *Chiaroscuro* Extremes, we thus calculate the distance from the distribution of intensities in the canvas to distributions in which the intensities are at either minimal or maximal possible values. (In our study, these intensities are 0 and 255 respectively.) To measure this distance, we begin by obtaining the midway point between the minimal or maximal possible intensities. We then perform a sum with two components: 1. sum of the subtractions of the intensities below the midway point and the minimal possible intensity; 2. sum of the subtractions of the maximal intensity and the intensities above the midway point. This sum should be zero only if the canvas has the desired property. In contrast, this sum reaches a maximal value when the image is homogeneous at the mid gray. We then construct the Index of *Chiaroscuro* Extremes, which is linearly related with this sum, being 0 and 1 when the sum 1 and 0 respectively.

Let $I\left(p_{k,j}\right)$ be the intensity of the pixel $p_{k,j}$ in Row k and Column j of the image. Let the number of rows and columns be $N_{r}$ and $N_{c}$ respectively. (We ensure that $N_{c}$ is even). Consequently, the total number of pixels is $N=N_{r}N_{c}$. Finally, let $I^{*}$ be the maximal possible intensity (the minimum being 0).

Denote the set of all pixels in the image as $S=\left\{p_{k,j}\right\}$. Define two subsets of S, namely $S_{-}$ and $S_{+}$, with the following properties:

(6) $$S_{-}\cup S_{+}=S,\;S_{-}\cap S_{+}=\emptyset ,\;p_{k,j}\in S_{-}⟹I\left(p_{k,j}\right)<\frac{I^{*}}{2},\;p_{k,j}\in S_{+}⟹I\left(p_{k,j}\right)>\frac{I^{*}}{2}$$

Hence, $S_{-}$ and $S_{+}$ contain all pixels with intensities below and above $I^{*}/2$ respectively. (Because intensities are integers and $I^{*}/2=127.5$, $S_{-}$ and $S_{+}$ contain all the pixels in S.) We use Equation (6) to define the Index of *Chiaroscuro* Extremes through the sum:

(7) $$C_{E}=\underset{p\in S_{-}}{\sum }I\left(p\right)+\underset{p\in S_{+}}{\sum }\left(I^{*}-I\left(p\right)\right)$$

This sum would have an upper bound at $NI^{*}/2$, if the image were homogeneous with intensity $I^{*}/2$, the middle gray. We thus define the Index of *Chiaroscuro* Extremes as:

(8) $$i_{c_{E}}=1-\frac{2C_{E}}{NI^{*}}$$

Therefore, $0\leq i_{c_{E}}\leq 1$. The value of $i_{c_{E}}$ would be 0 if the image were homogeneous with intensity $I^{*}/2$, and would be 1 if all the pixels of the image were black or white.

A possible limitation of the Index of *Chiaroscuro* Extremes was that some images could be entirely very dark or entirely very bright, and the index would still suggest the presence of *chiaroscuro*. If for example, an image was homogeneously back, the Index of *Chiaroscuro* Extremes would be 1. Although this limitation was unimportant for images of Renaissance portrait paintings, we decided to devise an alternate index of *chiaroscuro*. The new index measured how different the highest and lowest intensities were. To make this measurement, we again divided the set of points in the image into two sets. One set had all the points with intensities above a given percentile, while the other had the points with intensities below this percentile. We then subtracted the median intensity in the dark set from the median intensity in the bright set. As the result of this subtraction increases, we have more evidence of *chiaroscuro*. Thus, we use the result of this subtraction to construct an Index of *Chiaroscuro* Intensities. This index is linearly related to the result of the subtraction. The index is 0 when the subtraction yields 0, and 1 when the subtraction is equal to the difference between the maximal and minimal possible intensities. The higher this index is, therefore, the higher is the *chiaroscuro* intensity difference.

We again split S into two subsets, but this time based on a given percentile. We define $S_{-,f}$ and $S_{+,f}$ with the following properties:

(9) $$S_{-,f}\cup S_{+,f}=S,\;S_{-,f}\cap S_{+,f}=\emptyset ,\;\left|S_{-,f}\right|=fN,\;\left|S_{+,f}\right|=\left(1-f\right)N,\;p_{k,j}\in S_{-,f},\;p_{l,m}\in S_{+,f}⟹I\left(p_{k,j}\right)\leq I\left(p_{l,m}\right),$$

where 0≤f≤1, i.e., a fraction of 1. Hence, $S_{-,f}$ contains fN elements below the fth percentile of S and $S_{+,f}$ contains all the (1−f)N elements above the fth percentile of S. We use Equation (9) to define the Index of *Chiaroscuro* Intensities through the subtraction:

(10) $$C_{\Delta }={\tilde{S}}_{+,f}-{\tilde{S}}_{-,f},$$

where the wiggles denote medians. The result of this subtraction reaches its maximum, $I^{*}$, when the medians of ${\tilde{S}}_{+,f}$ and ${\tilde{S}}_{-,f}$ are the extremes, i.e., $I^{*}$ and 0 respectively. We thus define the Index of *Chiaroscuro* Intensities as:

(11) $$i_{c_{\Delta }}=\frac{C_{\Delta }}{I^{*}}$$

Consequently, $0\leq i_{c_{\Delta }}\leq 1$. The value of $i_{c_{\Delta }}$ would be 0 if ${\tilde{S}}_{+,f}={\tilde{S}}_{-,f}$, i.e., the intensities at the top and the bottom were similar, and would be 1 if ${\tilde{S}}_{+,f}=I^{*}$ and ${\tilde{S}}_{-,f}=0$, i.e., the intensities at the top and the bottom are maximally different.

### 2.6. Brief Descriptions of Symmetry, Balance, & Complexity

Elsewhere, we developed indices to quantify symmetry, balance, and complexity with methods and arguments like those in Section 2.5 [4]. Here, we describe these indices briefly to give the reader an intuitive understanding.

#### 2.6.1. Symmetry

Our measure of symmetry is bilateral. This measure is taken as a comparison of intensities of pixels equidistant from the vertical midline of the whole image. The difference between a pair of pixels can range from 0 (perfectly symmetric) to 255 (highly asymmetric). To compute our final measure, we take the root mean square of all of the pixel-wise computations and normalize by the maximum intensity. The result is a measure of asymmetry ranging from 0 to 1, which is what we use in all of our analyses.

#### 2.6.2. Balance

Like symmetry, we calculate our measure of balance across the vertical midline of the whole image. However, unlike symmetry, the computation for balance involves the integrals of the two sides rather than being pixel by pixel. The left and right integrals are then subtracted, and the absolute value of the result normalized by their sum. This measure gives an index of imbalance also ranging from 0 (full balance) to 1 (full imbalance).

#### 2.6.3. Thickness of Balance Line

We further extend the balance measure above to catch finer details of balance composition. Artists often compose an image by parts as well as a whole. For example, while an image may be perfectly balanced at the bottom of the canvas, it may not be at the top. The overall balance measure in Section 2.6.2 does not capture this difference. To do so, we take the same computation of balance but in a row-by-row manner. This gives us a row-wise vertical balance line. We measure the “thickness” of this line as the relative median absolute deviation of all the points on the line divided by the horizontal size of the canvas. Thus, this measure expresses the thickness of the balance line as fraction of the size of the canvas, and thus being similar for small and large paintings. The measure is such that the thicker the line is, the greater is the amount of changes in balance across the image.

#### 2.6.4. Complexity of Order 1

This form of complexity is the relative intensity entropy of an image. Thus, Complexity of Order 1 is the entropy of the distribution of intensities normalized by the maximal possible intensity entropy for an image of the same dimensions. An image with a wider distribution of intensities leads to greater Complexity of Order 1. This measure gives an index of complexity ranging from 0 (no complexity—only one intensity) to 1 (maximal complexity—all possible intensities equally likely).

#### 2.6.5. Complexity of Order 2

This form of complexity captures Complexity of Order 1 minus the loss of complexity due to the spatial organization of the image. While the distribution of pixel intensities may be the same in two images, the relative spatial organization can be different. For example, an image with relatively large regions of isometric intensities (for example, large objects, shadows, or walls) will be less complex due to the spatial grouping. In contrast, images with finer details (for example, embroidered clothing or smaller objects) will have greater complexity. In the latter example, Complexity of Order 2 will be larger than in the former. We measure the spatial organization underlying Complexity of Order 2 through the ability to explain the image with isometric transformations. (They are translations, rotations, reflections, and their compositions). Consequently, the resulting entropy comes from the two-dimensional distribution of intensities obtained by juxtaposing an image with all possible isometric transformations of it. We then divide the outcome by the maximal possible Entropy of Order 2 for an image of the same dimensions. Therefore, Complexity of Order 2 ranges from 0 to 1. An index of 0 for Complexity of Order 2 occurs in single-intensity images, while 1 happens for spatially random images with all possible intensities equally likely.

#### 2.6.6. Spatial Simplicity

As explained in Section 2.6.5, Complexity of Order 2 is Complexity of Order 1 minus the loss of complexity due to the spatial organization of the image. Hence, Complexity of Order 2 depends and is never greater than Complexity of Order 1. To obtain an index that captures spatial organization independently of Complexity of Order 1, we subtract from it Complexity of Order 2. We call this index Spatial Simplicity. To understand why, consider that the more spatially organized is the image (large homogeneous regions) the larger is Spatial Simplicity. From the definitions of Complexities of Order 1 and 2, Spatial Simplicity ranges from 0 to 1.

## 3. Results

### 3.1. Evolution of Neuroaesthetic Variables Throughout the Renaissance

**Figure 1:**
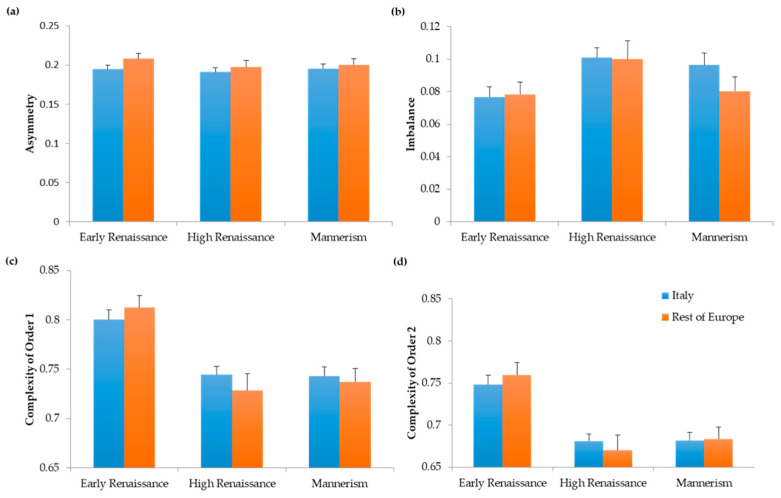
Spatiotemporal Evolution of Neuroaesthetic Variables throughout the Renaissance. (**a**) Index of Asymmetry; (**b**) Index of Imbalance; (**c**) Index of Complexity of Order 1; (**d**) Index of Complexity of Order 2. Error bars are standard errors. Whereas asymmetry remained statistically constant throughout the Renaissance, imbalance rose and complexities fell, especially in the Early Renaissance.

In this study, we were interested in the drift of values of neuroaesthetic variables in relation to the passing of time and the evolution of art. Because specialized brain mechanisms constrain these variables, one may expect that they remain relatively constant over time. In contrast, a recent study demonstrated that a certain degree of flexibility appears to exist with respect to neuroaesthetic variables [4]. Therefore, they could potentially evolve across different periods of art. We thus asked whether changes across art periods can occur in the absence of evolution of neuroaesthetic variables. To answer this question, we first performed computational measurements of asymmetry, imbalance, and complexity (normalized entropy—[3]). In particular, we probed the changes that happened to these variables in Italy and in the rest of Europe. Our study involved a time span of close to 200 years of Renaissance. In Figure 1, we see the results divided to the periods of Early Renaissance, High Renaissance, and Mannerism.

Our analysis revealed no significant changes in symmetry across the Early Renaissance, High Renaissance, and Mannerism (Figure 1a). In contrast, imbalance rose significantly between the Early and High Renaissance (Figure 1b, two-way ANOVA and post-hoc two-sided *t*-test, 298 d.f., t = 3.19, *p* < 0.002). The degree of imbalance grew by almost 30% in the span of 80 years. Complexity (i.e., normalized entropy) also evolved over time. We found falls in Complexities of Order 1 and Order 2 between the Early and High Renaissance (Figure 1c,d). These falls were significant for both Order 1 (432 d.f., t = 6.90, *p* < 2 × 10^−11^) and Order 2 (449 d.f., t = 7.26, *p* < 2 × 10^−12^). The falls reduced complexities of both orders by about 10%. Interestingly, however, no changes occurred in imbalance or complexity from High Renaissance to Mannerism. Hence, all changes in neuroaesthetic variables took place during the Early Renaissance. Finally, although we detected temporal changes in these variables throughout the Renaissance, we found no significant differences between Italy and the rest of Europe.

In conclusion, although symmetry was constant throughout the Renaissance, balance and complexities fell during the Early Renaissance.

### 3.2. Abrupt Transitions at the End of the 15th Century

**Figure 2:**
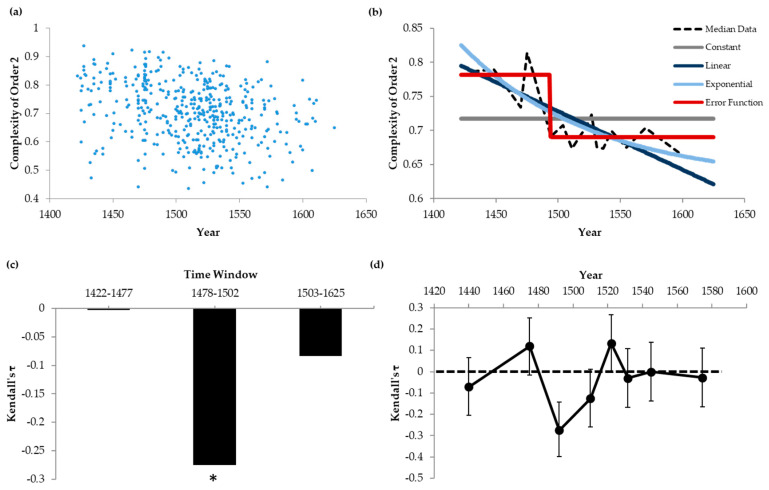
Analysis of the Scatter Plot of Complexities of Order 2 for Renaissance Paintings (**a**) Scatter Plot for All paintings (**b**) Best Fits of Four Models to Medians of the Data (25 paintings per median) (**c**) Kendall’s τ in Three Non-overlapping Time Windows During the Renaissance. The star indicates a Kendall’s τ statistically significantly different from zero (**d**) Kendall’s τ and their standard errors for non-overlapping, consecutive periods with 55 paintings each. The Complexity of Order 2 appeared to fall abruptly in the last decade of the 15th century.

To quantify these results further and to compare top artists with the other painters in our dataset, we produced scatter plots of the data. An example of the analysis of these plots appears in Figure 2 for Complexity of Order 2 (normalized spatial entropy).

The basic scatter plot for Complexity of Order 2 appears in Figure 2a, in which each point represents an individual painting. The abscissas correspond to the years of painting completion and the ordinates are the measured complexities. The data show great variability in each moment of portrait evolution. To quantify the variability, we calculated the ratio between the standard deviation and the mean of the Complexity of Order 2. Overall, this variability ratio was 15%. Despite the variability, the results in Figure 2a confirmed and extended the temporal trends in Figure 1. One observes that the Complexity of Order 2 falls during the Renaissance. The Kendall’s τ correlation coefficient was statistically significantly negative for Complexity of Order 2 (Kendall’s τ = −0.211, *p* < 3 × 10^−11^).

We attempted to characterize the fall of Complexity of Order 2 throughout the Renaissance by fitting four models (Equations (1)–(4); Figure 2b). Figure 1d had suggested that this fall was nonlinear and thus, we attempted exponential and error-function fits (Equations (3)–(5)). The latter seemed especially relevant, because no fall was apparent from the High Renaissance to the Mannerism. For completeness, we also attempted constant and linear fits. All the fits were statistically robust, by first extracting median complexities in small sections of the data (black dashed line in Figure 2b—obtained from the scatter plot with medians from 25 paintings).

The median Complexity-of-order-2 curve appeared to exhibit an abrupt fall around 1490. Not surprisingly, therefore, the Error-function model (Equation (4)) provided the best fit to the data. For example, the sums of squared errors for the optimal fits were 0.036, 0.014, 0.013, and 0.0068 for the Constant, Linear, Exponential, and Error-function models respectively. However, that the fit was better for the Error-function model was perhaps not surprising, because it had more parameters and could subsume some of the other models. Hence, we tested the quality of the fits with a regression test for arbitrary fits (Section 2.4; [21,22]). This test considered the number of parameters of the models. The test first plotted model predictions against the data and then analyzed the statistics of the resulting linear regression. The predictions were of positive correlation, with an intercept of 0 and a slope of 1. For all models, except the Constant one, we could not reject the null hypothesis that the correlation was positive. But the correlation was highest for the Error-function model ($R^{2}=0,\;0.61,\;0.65$, and 0.81 for the Constant, Linear, Exponential, and Error-function models respectively). Furthermore, we could reject that the intercept was zero for the Constant, Linear, and Exponential models (*p* < 0.0001, *p* < 0.005, and *p* < 0.008 respectively). In contrast, we could not reject this null hypothesis for the Error-function fit. Similarly, although we could not reject that the slope was 1 for the Error-function model, we could reject this null hypothesis for the Constant, Linear, and Exponential models (*p* < 0.0001, *p* < 0.005, and *p* < 0.008 respectively). Consequently, the Error-function model provided a superior fit than did the others. This superiority was true for all the fits in this article for data exhibiting trends. Moreover, we could not reject the Error-function model for any of these data.

The excellent error-function fit reinforced the conclusion of an abrupt fall of Complexity of Order 2 around 1490. The optimal transient year parameter ($t_{0}+\alpha_{3}$ in Equation (4)) was 1493. In addition, the optimal transition was indeed abrupt as shown by the red line Figure 2b. However, the transition was not as abrupt as suggested by the red line. This line was obtained by fitting the curve of medians from 25 paintings, corresponding to a span of 12 years around 1493, namely [1488–1500]. Therefore, all that we could say was that 12 years was the upper bound for the duration of the transition. We call this time window ([1488–1500] for this example) the upper-bound transition interval.

Such an abrupt transition was surprising, because it was not immediately apparent in the scatter plot (Figure 2a). We thus wished to obtain model-independent evidence for such a transition. This evidence is what Figure 2c,d show. In Figure 2c, we show the Kendall’s τ correlation coefficients for three non-overlapping section of the data. The middle section has 50 paintings around 1493. The other sections have all the paintings before and after the middle section. As the figure shows, although the middle section is far smaller than are the others, only it has a statistically significantly negative Kendall’s τ. The Kendall’s τ for the middle section is −0.275, with *p* < 0.004 that the correlation coefficient is 0. Comparison of this Kendall’s τ with that obtained for the entire data (−0.211) suggests that most of the fall of Complexity of Order 2 happens during the period encompassed by the middle section [1478–1502]. This result is compatible with the upper-bound transition interval estimated above.

Further confirmation of the conclusion of abrupt transition appears in Figure 2d. This figure plots the Kendall’s τ’s and their standard errors for non-overlapping, consecutive sections of the data comprising 55 paintings. Only one point in the plot is statistically significantly negative, namely, the one centered on 1492.

In conclusion, our data indicate an abrupt fall in Complexity of Order 2 in the last decade of the 15th century. Similar analyses have shown abrupt transitions in most other variables studied in this paper, except as indicated.

### 3.3. Dynamics of Neuroaesthetic Variables and the Top Painters

**Figure 3:**
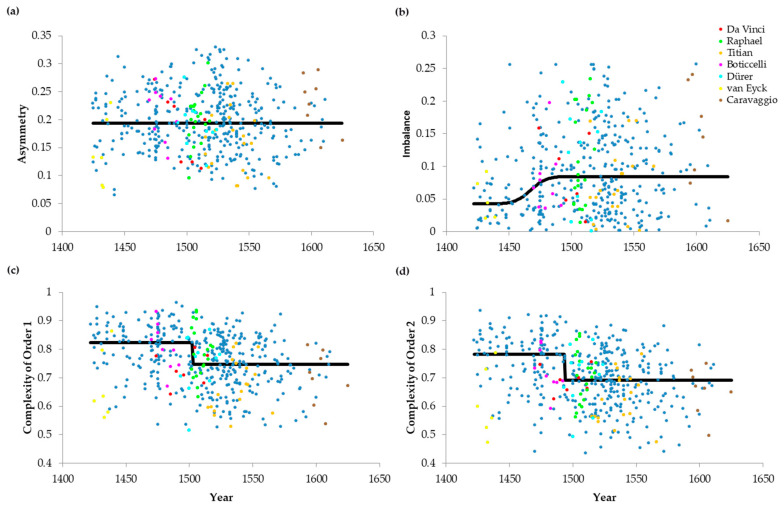
Scatter Plots of the Evolution of Neuroaesthetic Variables throughout the Renaissance with Error-function Fit (**a**) Index of Asymmetry (**b**) Index of Imbalance (**c**) Index of Complexity of Order 1 (**d**) Index of Complexity of Order 2. Data points from portraits of the top painters of the Renaissance are colored for ease of identification. Except for asymmetry, all aesthetic variables considered here undergo an abrupt transition at the end of the 15th century. For the most part, the statistics for the top painters are like those of their peers.

We extended the scatter-plot analysis of Section 3.2 to Asymmetry, Imbalance, and Complexity of Order 1. In particular, we superimposed on the scatter plots temporal trend lines to help quantify the time courses of the drift of values of neuroaesthetic variables (Equations (4) and (5)). Finally, we colored the points of the top artists (Section 2.2) to compare them with peers from their periods. The results appear in Figure 3.

As for Complexity of Order 2 (Figure 2), Figure 3 shows great variability in each moment of portrait evolution. This variability holds for the results of both the whole group of painters and the work of the leading masters. The ratio between the standard deviation and the mean is 28%, 73%, and 13% for asymmetry, imbalance, and Complexity of Order 1 respectively. Consequently, the variability is lowest for complexities (see also Section 3.2) and highest for balance.

Also, like Figure 2, despite the variability, the results in Figure 3 confirmed and extended the temporal trends in Figure 1. The Kendall’s τ correlation coefficient was not significantly different from zero for asymmetry (see its robust constant regression in Figure 3a). However, the coefficient was statistically significantly positive for Imbalance (Kendall’s τ = 0.0738, *p* < 0.03). In contrast, the coefficient was statistically significantly negative for Complexity of Order 1 (Kendall’s τ = −0.204, *p* < 2 × 10^−10^), as it was for Order 2 (Section 3.2). The Error-function regression lines (Equation (4)) attempted to capture these positive and negative tendencies in the evolution of the neuroaesthetic variables. The line rose for imbalance (Figure 3b; obtained from the scatter plot with medians from 30 paintings). But the line fell for complexities (Figure 3c,d; obtained from the scatter plot with medians from 25 paintings). The best-fit transition times for Imbalance and Complexities of Order 1 were 1467 and 1502 respectively. The corresponding upper-bound transition intervals (see discussion of Figure 2b) were [1451–1473] for imbalance and [1499–1505] for Complexity of Order 1. Therefore, the transition was about 25 years earlier for imbalance than Complexity of Order 2 (see also Section 3.2). The transition was also slower for imbalance. Moreover, the transition may have been about 10 years later for Complexity of Order 1 than of Order 2.

The top painters did not generally appear to behave differently from their peers in terms of neuroaesthetic variables. Statistical comparisons of the values of their neuroaesthetic variables show relatively equal distribution above and below the trend lines. Titian was the only exception for Complexity of Order 1 (*t* = 3.48, 17 d.f., and *p* < 0.003). In turn, two painters were exceptions for Complexity of Order 2. They were van Eyck (*t* = 3.42, 5 d.f., and *p* < 0.02) and Titian (*t* = 3.11, 17 d.f., and *p* < 0.007). These painters produced portraits that were less complex than were those of the peers, as evaluated by the Error-function model.

In sum, balance and complexity declined abruptly towards the end of the 15th century, but this fall was not generally due to the top painters of those times. Together with the results of Section 3.1 and Section 3.2, we thus established that neuroaesthetic variables were not constant throughout the Renaissance.

### 3.4. Evolution of the Thickness of Balance Lines

**Figure 4:**
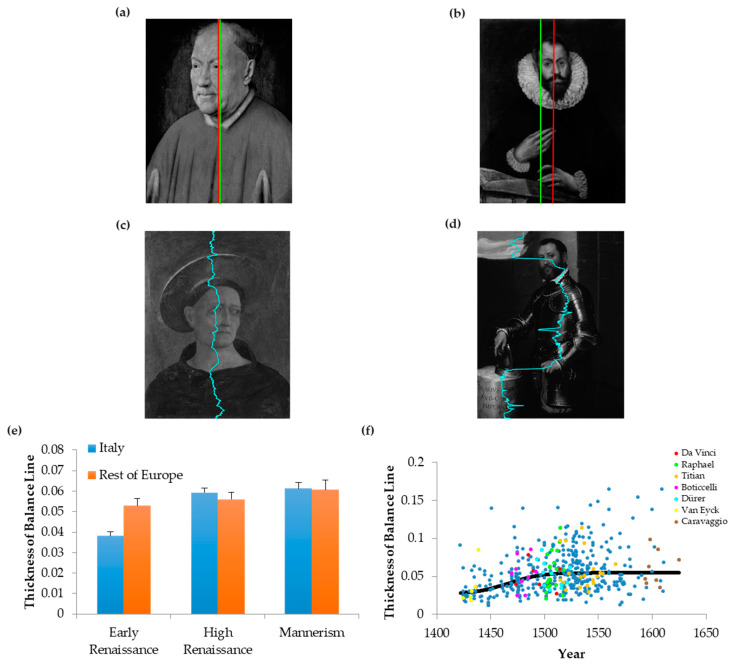
Balance and Balance Line. (**a**,**b**) Portraits with Midline (Red) and Position of Balance (Green) Marked. (**a**) Jan van Eyck, Portrait of Cardinal Niccolo Albergati Papal Envoy in the Spanish Netherlands, 1431–1432, Kunsthistorisches Museum, Viena, Austria. Photo Credit: Erich Lessing/Art Resource, N.Y. (**b**) El Greco, Portrait of a Man (possibly Alonso de Herrera) 1600, Musée de Picardie, Amiens and Picardy, France. (**c**,**d**) Portraits with Balance Lines Marked. (**c**) Domenico Veneziano, Head of a Tonsured, Beardless Saint 1440-4, The National Gallery, London, Great Britain. Photograph: ©The National Gallery, London National Gallery Picture Library, The National Gallery Company. (**d**) Giovanni Battista Moroni; Portrait of Mario Benvenuti 1560, The John and Mable Ringling Museum of Art, the State Art Museum of Florida, a division of Florida State University. (**e**) Spatiotemporal Evolution of the Thicknesses of Balance Lines throughout the Renaissance. (**f**) Scatter Plot of the Evolution of the Thicknesses of Balance Line throughout the Renaissance. Conventions for Panels E and F are as in Figure 1 and Figure 3. The thickness of balance line was low in Italy during the Early Renaissance, but rose before the High Renaissance.

To understand the decline of balance over time, we must start from the definition of imbalance. We defined it relative to the midline of the canvas. Elsewhere, we also considered the position of balance, i.e., the place for which the integrals of intensities to the right and left of it were equal [4]. Thus, the decline of balance in the Renaissance meant that the distance between the midline and the position of balance tended to increase over time (see examples in Figure 4a,b). However, this decline did not imply a rise in the sloppiness of balancing different parts of the portrait. Painters could continue to balance portraits delicately but simply do it in a position of balance away from the midline. In an earlier publication, we reported that painters in the Early Renaissance not only balanced their portraits, but also did so at every row of the canvas [4]. We thus decided to test if this delicate form of balance was also diminished as the Renaissance progressed. To do so, we measured the positions of balance at every height of the painting. All these points together formed the Balance Line (Figure 4c,d). We then measured the thickness of this line as a fraction of the horizontal size of the canvas. The results appear in Figure 4e,f.

The results show that the thickness of the balance line also rises in the Renaissance. We can appreciate an example by comparing a portrait by Domenico Veneziano in the Early Renaissance with one by Giovanni Battista Moroni during Mannerism (Figure 4c,d, respectively). In Veneziano’s portrait, the balance line shows that the distributions of intensities on the two sides of the midline of the canvas are similar. The balance line is close to midline at every height analyzed. In contrast, in Moroni’s portrait, the balance line has more variation across vertical positions. Hence, the balance line in Moroni’s portrait has more thickness (0.165) than in Veneziano’s (0.018). Thus, Moroni was “sloppier” in balancing different parts of his portrait than was Veneziano.

This difference held when we analyzed the thicknesses of balance lines throughout the Renaissance. In the Early Renaissance, the thickness of the balance line was significantly lower in Italy than in the rest of Europe (two-way ANOVA and post-hoc two-sided post-hoc *t*-test, 149 d.f., *t* = 4.05, *p* < 9 × 10^−5^—Figure 4e).

Afterwards the thickness of the balance line grew in Italy from the Early to High Renaissance (211 d.f., *t* = 7.00, *p* < 4 × 10^−11^—Figure 4e), catching up with the values in the rest of Europe. In contrast, the thickness of the balance line was statistically constant in the rest of Europe throughout the Renaissance. Therefore, portraits in the rest of Europe were more prescient of future trends of balance than were Italian ones.

The scatter plots showed that the thicknesses of the balance lines (Figure 4f) followed a trend like that of imbalance (Figure 3d). The data in Figure 4f show great variability of thicknesses in each moment of portrait evolution. The ratio between the standard deviation and the mean of the thicknesses of balance lines was 50%. Despite the variability of thicknesses, the results in Figure 4f confirmed and extended the temporal trends in Figure 4e. Portraits tended to be carefully balanced in the Early Renaissance, but exhibit sloppier balances in the High Renaissance and Mannerism. Accordingly, the Kendall’s τ correlation coefficient was statistically significantly positive for the thicknesses of balance lines (Kendall’s τ = 0.190, *p* < 1.21 × 10^−9^). The best-fit transition time was 1467, confirming that most change happened in the Early Renaissance. However, the change was much slower for the thickness of balance line than for other aesthetic variables. Consequently, its change was not abrupt. Finally, as for imbalance (Figure 3b), top painters did not generally produce portraits with thicker balance lines than those of peers (Figure 4f). In conclusion, as the Early Renaissance progressed, painters tended to become “sloppier” in balancing different parts of the portrait.

### 3.5. Evolution of Spatial Complexity

**Figure 5:**
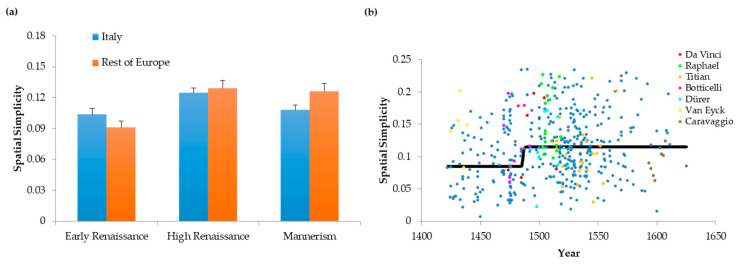
Evolution of Spatial Simplicity throughout the Renaissance. (**a**) Spatiotemporal Evolution. (**a**) Scatter Plot of the Evolution. Conventions for Panels (**a**) and (**b**) are as in Figure 1 and Figure 3. Spatial simplicity was lower in the Early Renaissance, but rose abruptly at the end of the 15th century.

How are we to understand the decline of complexity over time? The Complexities of Order 1 and 2 in Figure 1 and Figure 3 have different types of interpretation [4]. Complexity of Order 1 measures the normalized entropy in the distribution of intensities in the image. In turn, Complexity of Order 2 begins from Complexity of Order 1 and then discounts the reduction of entropy due to spatial organization. Consequently, if we want to isolate the loss of complexity due to spatial organization alone, we must calculate Complexity of Order 1 minus Complexity of Order 2. We call this quantity the Spatial Simplicity [4]. The temporal drift of the Spatial Simplicity throughout the Renaissance appears in Figure 5.

Portrait paintings tended to become spatially simpler as the Renaissance progressed. Thus, in High Renaissance and Mannerist periods, spatial complexity was lower than in the Early Renaissance (Figure 5a; *t* = 4.38, 447 d.f., *p* < 0.00002). However, as for Figure 1, although we detected temporal changes in Spatial Simplicity throughout the Renaissance, we found no significant differences between Italy and the rest of Europe. The scatter plots confirmed the rise of spatial simplicity (Figure 5b). Accordingly, the Kendall’s τ correlation coefficient was statistically significantly positive for spatial simplicity (*τ* = 0.0994, *p* < 0.002). The Error-function regression lines (Equation (4)) rose abruptly for Spatial Simplicity (Figure 5b; obtained from the scatter plot with medians from 35 paintings). The best-fit transition time was 1486, with the upper-bound transition interval lasting 17 years, namely, [1477–1494]. Finally, four of the seven top painters produced portraits with different spatial-simplicity distributions than those of their peers (Figure 5b). Botticelli (*t* = 2.55, 8 d.f., *p* < 0.04), van Eyck (*t* = 3.19, 4 d.f., *p* < 0.04), and Raphael (*t* = 3.76, 18 d.f., *p* < 0.002) exhibited more spatial simplicity than did their peers. In contrast, Caravaggio exhibited less (*t* = 3.60, 7 d.f., *p* < 0.009).

Hence, spatial complexity (the component of entropy due to spatial organization) fell abruptly towards the end of the Early Renaissance. This fall mimicked the decline of the complexity due to the distribution of intensities (Complexity of Order 1—Figure 3c).

### 3.6. Chiaroscuro and the Fall of Complexity

**Figure 6:**
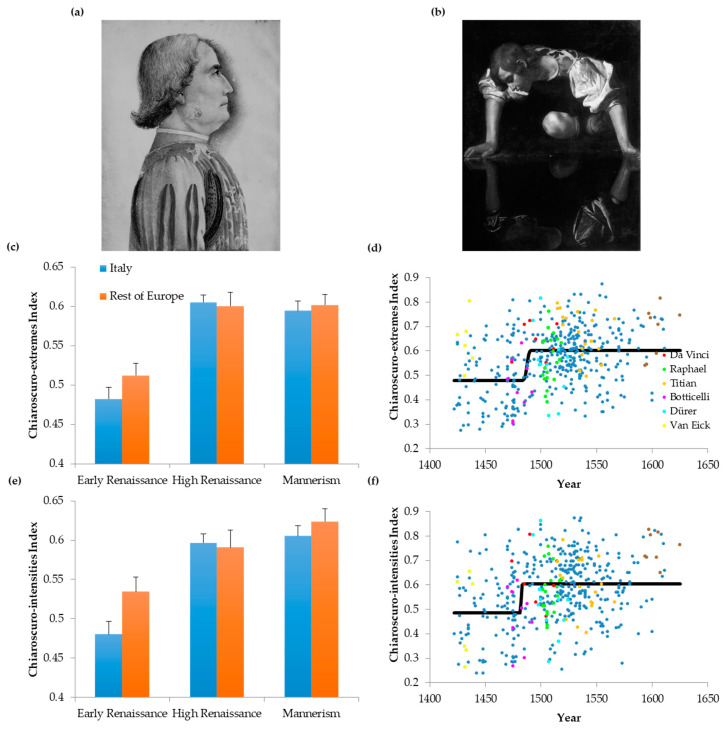
Evolution of Chiaroscuro. (**a**,**b**) Illustration of Portraits without (**a**) and with (**b**) chiaroscuro. (**a**) Andrea Mantegna, Portrait of Jacopo Antonio Marcello, 1453, Bibliothèque de l’Arsenal, Paris, France. Photo Credit: Erich Lessing/Art Resource, N.Y. (**b**) Michelangelo Merisi da Caravaggio, Narcissus, 1597, Galleria Nazionale D’arte Antica nel Palazzo Corsini, Rome, Italy © 2006, Scala, Florence/Art Resource, N.Y. C. Spatiotemporal Evolution of the Index of Chiaroscuro Extremes. (**d**) Scatter Plot of the Evolution of the Index of Chiaroscuro Extremes. (**e**) Spatiotemporal Evolution of the Index of Chiaroscuro Intensities. (**f**) Scatter Plot of the Evolution of the Index of Chiaroscuro Intensities. Conventions for Panels A and B are as in Figure 1 and Figure 3. The degree of chiaroscuro rose abruptly in the Early Renaissance.

The decline of complexity over time (Figure 1c,d, Figure 2, Figure 3c,d and Figure 5) was surprising to us. We had expected complexity to increase as paintings became more realistic in the Renaissance. Ideas that evolved throughout the Renaissance, such as naturalism, anatomical studies, linear perspective, and aerial perspective should perhaps have made portraits more complex. Therefore, we wondered why complexity fell. We hypothesized that portrait paintings got simpler with the invention of *chiaroscuro.* It introduced large dominant regions with fewer colors and homogeneous intensities. We can appreciate an example of such regions by comparing a portrait by Andrea Mantegna in the Early Renaissance with one by Caravaggio during Mannerism (Figure 6a,b, respectively). Mantegna’s portrait has no dominant regions in terms of blacks and white, and thus has no or very little *chiaroscuro*. In contrast, Caravaggio’s portrait is a good example of *chiaroscuro*, with some bright regions contrasting against a large, dark background. Hence, the indices of *chiaroscuro* extremes (Equation (8)) and *chiaroscuro* intensities (Equation (11)) in Mantegna’s portrait (0.33 and 0.094 respectively) were lower than those in Caravaggio’s (0.75 and 0.83 respectively). Thus, our measurements confirm the common knowledge that Caravaggio used *chiaroscuro* more than did Mantegna (and most other painters—[8]). A quantitative study of these *chiaroscuro* indices across the Renaissance appears in Figure 6c–f.

Figure 6c revealed that the index of *chiaroscuro* extremes (Equation (8)) rose between the Early Renaissance and the High Renaissance periods (two-way ANOVA and post-hoc two-sided *t*-test, 303 d.f., *t* = 8.28, *p* < 4 × 10^−15^). However, no such rise occurred from the High Renaissance to Mannerism. Figure 6d also showed that this index grew abruptly towards the end of the Early Renaissance (Kendall’s τ = 0.231, *p* < 3 × 10^−13^; best-fit transition time = 1487, upper-bound transition interval lasting 15 years, namely, [1479, 1494]). These findings were replicated for the index of *chiaroscuro* intensities (Equation (11)) in Figure 6e,f (313 d.f., *t* = 5.92, *p* < 9 × 10^−9^; Kendall’s τ = 0.194, *p* < 8 × 10^−10^; best-fit transition time = 1481, transition interval lasting 14 years, namely, [1476, 1490]). Another finding in Early Renaissance was the statistical similarity of Italy with the rest of Europe in terms of *chiaroscuro* tendencies. Finally, we again detected little difference from top painters and their peers (Figure 6d,f). The only exceptions were van Eyck for the index of *chiaroscuro* extremes (*t* = 3.75, 5 d.f., *p* < 0.02), and Caravaggio for the index of *chiaroscuro* intensities (*t* = 6.67, 7 d.f., *p* < 3 × 10^−4^). Both van Eyck and Caravaggio exhibited more chiaroscuro than did their peers.

**Figure 7:**
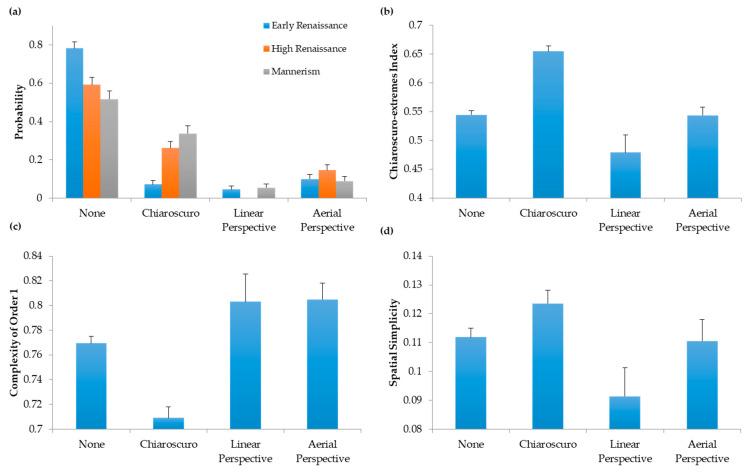
Link between Chiaroscuro and Complexity. (**a**) Evolution of Various Compositional Elements throughout the Renaissance. (**b**) Link between these Compositional Elements and the Index of Chiaroscuro Extremes. (**c**) Link between these Compositional Elements and Complexity of Order 1. (**d**) Link between these Compositional Elements and Spatial Simplicity. Error bars are standard errors. The emergence and rise of chiaroscuro accounted for the fall of complexity in the Renaissance.

That the degree of *chiaroscuro* usage went up in the Renaissance was consistent with our hypothesis for the decline of complexity. However, we still had to demonstrate that more *chiaroscuro* in a portrait tended to lead to less complexity. In Figure 7, we classified portrait paintings in three compositional concepts that could affect complexity: linear perspective, aerial perspective, and *chiaroscuro* (Section 2.3). We also included a class for those portraits that do not belong to any of these three categories. Finally, we quantified Complexity of Order 1, spatial simplicity, and the index of *chiaroscuro* extremes in these four categories.

In Figure 7a, we observe that the prevalence of *chiaroscuro* portraits increases as time progresses in the Renaissance. This increase occurs specially from the Early to the High Renaissance (Fisher’s exact test, odds ratio = 0.30, *p* < 5 × 10^−4^). Although we categorized these portraits by hand (Section 2.3), Figure 7b supported the idea that our *chiaroscuro* category was correct. The index of *chiaroscuro* extremes was higher for this category than was for the others (one-way ANOVA followed by post-hoc one-sided *t*-tests; linear perspective, 114 d.f., *t* = 6.37, *p* < 3 × 10^−9^; aerial perspective, 151 d.f., *t* = 6.52, *p* < 6 × 10^−10^; None, 381 d.f., *t* = 8.28, *p* < 2 × 10^−15^). Consequently, the use of *chiaroscuro* techniques increased over time. In contrast, the prevalence of portraits with the other tested compositional categories, namely, linear and aerial perspective, was statistically constant throughout the Renaissance. Hence, of the compositional elements studied, *chiaroscuro* is the only candidate available to explain the fall of complexity over time. Is *chiaroscuro* contributing to the simplification of portraits? The answer to this question appears in Figure 7c,d. The former shows that Complexity of Order 1 is significantly lower in portrait paintings with *chiaroscuro* than in portraits in the other categories (linear perspective, 106 d.f., *t* = 3.83, *p* < 2 × 10^−4^; aerial perspective, 143 d.f., *t* = 6.09, *p* < 5 × 10^−9^; None, 378 d.f., *t* = 5.40, *p* < 6 × 10^−8^). Consequently, *chiaroscuro* reduces the complexity of the distribution of intensities. Furthermore, in Figure 7d, we see that spatial simplicity is significantly lower in portraits with *chiaroscuro* than is in portraits of the other categories (linear perspective, 106 d.f., *t* = 3.30, *p* < 2 × 10^−3^; aerial perspective, 142 d.f., *t* = 2.57, *p* < 6 × 10^−3^; None, 372 d.f., *t* = 3.37, *p* < 5 × 10^−4^). Therefore, *chiaroscuro* tends to reduce the spatial complexity of portraits. We conclude that the reduction in complexity in the Renaissance may be due to the rise of *chiaroscuro*.

## 4. Discussion

### 4.1. Neuroaesthetic Variables Evolved Throughout the Renaissance

In the Introduction, we raised the hypothesis that specialized brain mechanisms might constrain neuroaesthetic variables to remain relatively constant across art periods. However, our analysis of the Renaissance ruled out this hypothesis, showing that neuroaesthetic variables evolved.

This evolution tended to be abrupt towards the end of the Early Renaissance (Figure 1, Figure 2 and Figure 3, Figure 5 and Figure 6). We observed abrupt transitions centered around 1465 for balance-related variables, around 1490 for spatial complexity and *chiaroscuro*, and around 1500 for intensity complexity. These different transition centers were significant, because their corresponding upper-bound transition intervals did not overlap. The transition was significantly slower for balance-related variables than for the others (see for example, Figure 4f). As for the other variables, the transitions were fast. They lasted less than about 15 years for spatial complexity and chiaroscuro, and no more than about 6 years for intensity complexity.

What are possible explanations for these abrupt transitions? The median data on Figure 2b and the model fits in Figure 3, Figure 5 and Figure 6 suggest that these abrupt changes correspond to phase transitions. Phase transitions are common in natural sciences (for example, the abrupt transition from ice to liquid water as a function of temperature), but may also occur in the social sciences [24]. In our data, the phases are demonstrated by the relatively constant median values of the aesthetic variables before and after the transitions. However, although the transition between the phases is fast, it is not discontinuous. Hence, the abrupt changes of aesthetic values are second-order phase transitions (like the magnetization in iron) and not first-order (like the ice-to-water transformation). The essential components of such transitions are nonlinear interactions between the basic components of a system, which is under the influence of changing external conditions. We propose that the basic elements are the values associated with different instantiations of aesthetic variables by individual artists. In turn, the nonlinear interactions are due to mutual influence between artists learning from each other [7]. Finally, the changing external environment could exert social pressure to innovate and lead to cultural trends ([25]—for example, the desire for increasing realism during the Renaissance—[8]). With such external pressure, the phase transitions of aesthetic variables could happen as follow: Under pressure to innovate, an artist invents a technique, whose aesthetic value is superior to extant pieces of art (for instance, *chiaroscuro*). A small number of other artists are exposed to the new technique, using and perfecting it. As the number of artists using the technique increase, the probability that others learn increases exponentially. Therefore, the use of technique accelerates rapidly, causing the phase transition.

### 4.2. What Explains the Unexpected Declines of Balance and Complexity

Why did balance decrease throughout the Renaissance? Because balance is highly salient and preferred [26,27,28], we might have expected balance to increase over time. An explanation for the decay of balance and the thickening of balance lines might be the rise of dynamism in paintings, leading to the Baroque period [8]. Representation of motion brings with itself new neuroaesthetic variables that might compete with balance. Such competition exists between complexity and balance [29]. Computer simulations with a new theory for the learning of aesthetic values suggest that learning under the influence of motivated behaviors may have a role in generating these competitions [7].

Why did complexity fall throughout the Renaissance? Our expectation before starting this study was that the addition of details in paintings should perhaps have increased their entropy and thus, complexity. After all, Renaissance painters were striving to make their portraits more realistic, by studying nature and adding features such as perspective [30]. Perhaps with the realism, complexity would rise. We considered different factors that could fight complexity. One factor would be the invention of aerial perspective, with its tendency to smear details from distant objects [8]. However, our results argued against the aerial-perspective factor (Figure 7). An alternate factor for the fall of complexity would be the gradual emergence of *chiaroscuro*, with its large dominant regions with relatively homogeneous colors or intensities. Our data confirm the relevance of this factor, showing a strong link between the fall of complexity and the rise of *chiaroscuro* (Figure 7). This link makes an interesting point: Fall in complexity does not imply fall in realism. Part of the reason to use *chiaroscuro* is to increase the sense of realistic three-dimensional space through shadow effects [31].

### 4.3. Temporal Constancy of Symmetry

Different from balance and complexity, we did not detect temporal trends in the degree of symmetry in portrait paintings across the Renaissance (Figure 1a and Figure 3a). However, this lack of trend did not mean that symmetry was frozen. We used the Kendall’s τ statistic to test for such trends. This statistic is used to measure the ordinal association between two quantities [16]. However, different aspects of the distribution of symmetries could have changed over time without affecting this association. For example, inspection of Figure 3a suggests a temporal change of the variance in the distribution of the Index of Asymmetry.

Why did the central tendency of symmetry not exhibit a temporal trend? The emphasis on symmetry appeared as a rebirth of the classical ideas of composition from antiquity [31]. Hence, perhaps the cultural force of the classic ideals kept symmetry strong throughout the Renaissance. An alternative is that symmetry is an important variable in the brain, thus constraining what painters do. We believe that this is not the case, because from the Early Renaissance, portrait painting is not mostly frontal, therefore with an automatic break of symmetry [32]. Instead, we propose that our mathematics of symmetry is delicate, because of the point-by-point comparisons. Consequently, the variability of the data could have destroyed temporal trends of symmetry. One component of the variability could have arisen by our choice of using the Artstor Digital Library. Therefore, the digital images were acquired by different institutions without common standards for color balance or lighting conditions. Future studies of temporal trends of symmetry should try to standardize the methods of image acquisition. Other factors of variability beyond image acquisition are individuality of artists, image degradation over time, and image restoration. Those factors are harder to control and may cause the permanent loss of any possible small temporal trends of symmetry. Fortunately, all these factors of variability do not invalidate statistically significant temporal trends such as those observed for balance, complexities, and chiaroscuro. This is especially fortunate for the latter, because degradation and restoration could have had a specially devastating effect on chiaroscuro trends.

In contrast to symmetry, the mathematics of balance and complexity involve integrations that make the measurement of these variables more robust. Consequently, variations across paintings could impair detection of any changes of symmetry that may be occurring during the Renaissance. Future efforts could try to bypass this potential limitation by using either multiple measures of symmetry (for example, radial—[33]) or object-wise, local symmetry [34,35] instead of global measures obtained from the whole picture.

### 4.4. Italy Versus the Rest of Europe

A surprising result in our study was obtained when comparing the evolution of balance in Italy versus the rest of Europe. When measuring the progression of the thickness of balance lines, the rest of Europe seemed to be more prescient about their future trends than Italy was. For example, the thicknesses of balance lines rose over time during the Renaissance (Figure 4e,f). These thicknesses were already larger in the rest of Europe than in Italy in the Early Renaissance (Figure 4e). Similar results (although without strong statistical significance) held for Spatial Simplicity and both indices of *chiaroscuro* (Figure 5a and Figure 6c,e). How could we understand these results given that for most scholars, Italy was the most influential site of the Renaissance [36]. In truth, the Northern Renaissance remained relatively independent of Italy until the end of the 15th century [8]. In addition, some scholars even suggest a North-to-South direction of influence [37,38]. Venetians had much in common with the Flemish in their oil technique and representation of light. Only after 1500, the Italian Renaissance began influencing the rest of Europe. Therefore, the rest of Europe could develop a style with both *chiaroscuro* and relatively low spatial complexity before did the Italian Renaissance.

### 4.5. Top Master Painters Versus Peers

We were curious whether the top master painters of the Renaissance distinguished themselves by having different values of neuroaesthetic variables. When we probed this issue, we found that for the most part, the top artists had similar statistics as the rest of their contemporaries. (The standardization of the methods of image acquisition proposed in Section 4.3 could help reveal more delicate differences between artists.) However, there were some interesting and important exceptions. One of the most important examples was van Eyck, who was ahead of his time in terms of various artistic trends of the Renaissance. He led specially in trends related to *chiaroscuro* and spatial complexity of the portraits (Figure 3d, Figure 5b and Figure 6d). These van Eyck results are compatible with those discussed in Section 4.4. We pointed out in that section that Flanders was ahead of Italy in terms of neuroaesthetic variables in the Early Renaissance. Another worthwhile painter to mention in terms of uniqueness of neuroaesthetic variables was Caravaggio, whose portraits showed high spatial complexity (Figure 5b). Thus, although he painted in *chiaroscuro* [8], the bright portions of his paintings were highly complex.

A possibly surprising negative result was that da Vinci’s portraits did not yield *chiaroscuro* indices statistically significantly higher than did those of his peers (Figure 6d,f). This is surprising, because many consider that in European painting, he was the one who brought the technique to its full potential [8,39]. He painted some clearly *chiaroscuro* pieces (for example, The Adoration of the Magi, 1481). However, he also had non-*chiaroscuro* portraits (for example, the Mona Lisa, 1517). Figure 6f illustrates this variety of da Vinci styles. This figure shows that his portraits yield indices of *chiaroscuro* intensities in roughly equal amounts above and below the fit line. This distribution contrasts sharply with that for Caravaggio, for whom the fit line is entirely below the corresponding indices.

Apropos Leonardo da Vinci’s paintings, a modification of our techniques may be able to reveal some special statistics as compared to his contemporaries. da Vinci is famous for his sfumato technique [10,27]. If we increased the spatial resolution of our analysis, we could perhaps gauge sfumato through Complexity of Order 2 in small translations of the image. However, we would have to perform this analysis near the edges of image objects. To achieve this goal, we would have to add shape analysis, such as edge detection to our study [40]. Such shape analysis could enhance our studies in the future in other ways. In our paper, the analysis of aesthetic variables was performed with global tools, accounting for pixel statistics obtained from the entire image. An interesting question concerns the analysis of these variables in shapes in the centerpiece. These shapes would be faces in the case of portraits. Using edge-detection and machine-learning algorithms [41]), the outlines of faces in paintings could be selected. Then, the same measures used in this paper could in principle be applied to these selections. Based on our previous study [4], we believe that these focused measures might reveal interesting results. In that study, we did a manual pose classification of the subjects of portrait paintings and found that artists seldom painted their subjects in frontal poses, instead opting for a side-on or ¾ pose. Often, the pose was such that the head would be turned in relation to the torso to create greater variation. Our analysis showed that those paintings with complex poses had greater Complexity of Order 2, and lesser balance and symmetry, which is what we would expect here as well.

### 4.6. Implications of Evolution across Art Periods

Why does the distribution of the values of neuroaesthetic variables drift over time if the brain constrains them? This is only possible if these variables have flexibility to change in the brains of artists (and other people). Elsewhere, we propose that the range of values of these variables form a space, which we termed neuroaesthetic space [4]. The aesthetic choices of each artist would reside in a sub-region of this space. The locations of this sub-region depend on both the life experience of each artist and the materials and techniques available him or her. Consequently, artists learn from their social and cultural background, and especially, from other artists of the cultural moment. Thus, we propose two principles as guides for how neuroaesthetic variables evolved in portrait painting throughout the Renaissance: (1) New materials, techniques, or ideas by artists, propelled other artists to change through learning from the cultural environment. In this paper, the best example was the evolution of chiaroscuro. Our data set contained a portrait from as early as 1438 that belonged to the chiaroscuro category (Portrait of Giovanni Arnolfini, Jan van Eyck). Other painters liking the result were compelled to produce more and better chiaroscuro pieces (Figure 7a). (2) The necessity of artistic innovation was accelerated by different workshops competing for the favor of patrons [12]. For example, many artists included in this study, such as Pollaiuolo, da Vinci, Botticelli, and Ghirlandaio competed for Lorenzo de’ Medici’s. Thus, such artists reciprocally affected each other’s artistic evolution, pushing aesthetic variables to evolve rapidly. Hence, the artistic influence of aesthetic variables may have evolved in a manner related to biological co-evolution [42].

## Figures and Tables

**Table 1:** Top Ten Renaissance Artists According to Ranker.com.

	Name	# of Votes	Period	In this Study
1	da Vinci	1322	High Renaissance	Yes
2	Michelangelo	1071	High Renaissance	No
3	Raphael	713	High Renaissance	Yes
4	Donatello	599	High Renaissance	No
5	Titian	413	High Renaissance	Yes
6	Botticelli	407	Early Renaissance	Yes
7	Caravaggio	296	Mannerism	Yes
8	van Eyck	275	Early Renaissance	Yes
9	Brunelleschi	258	Early Renaissance	No
10	Dürer	257	High Renaissance	Yes

## Supplementary Materials

The supplementary materials for this article are available online at https://github.com/ha554n/Neuroaesthetic-Variables-Measures.
